# It’s About Me: Patients’ Experiences of Patient Participation in the Web Behavior Change Program for Activity in Combination With Multimodal Pain Rehabilitation

**DOI:** 10.2196/jmir.5970

**Published:** 2017-01-18

**Authors:** Catharina Nordin, Peter Michaelson, Margareta K Eriksson, Gunvor Gard

**Affiliations:** ^1^ Department of Primary Health Care Region Norrbotten Piteå Sweden; ^2^ Department of Health Science Division of Health and Rehabilitation Luleå University of Technology Luleå Sweden; ^3^ Department of Public Health Region Norrbotten Luleå Sweden

**Keywords:** interview, pain, patient participation, qualitative research, Web-based intervention

## Abstract

**Background:**

Patients’ participation in their health care is recognized as a key component in high-quality health care. Persons with persistent pain are recommended treatments with a cognitive approach from a biopsychosocial explanation of pain, in which a patient’s active participation in their rehabilitation is in focus. Web-based interventions for pain management have the potential to increase patient participation by enabling persons to play a more active role in rehabilitation. However, little is known about patients’ experiences of patient participation in Web-based interventions in clinical practice.

**Objective:**

The objective of our study was to explore patients’ experiences of patient participation in a Web Behavior Change Program for Activity (Web-BCPA) in combination with multimodal rehabilitation (MMR) among patients with persistent pain in primary health care.

**Methods:**

Qualitative interviews were conducted with 15 women and 4 men, with a mean age of 45 years. Data were analyzed with qualitative content analysis.

**Results:**

One theme, “It’s about me,” and 4 categories, “Take part in a flexible framework of own priority,” “Acquire knowledge and insights,” “Ways toward change,” and “Personal and environmental conditions influencing participation,” were developed. Patient participation was depicted as being confirmed in an individualized and structured rehabilitation framework of one’s own choice. Being confirmed was fundamental to patient participation in the interaction with the Web-BCPA and with the health care professionals in MMR. To acquire knowledge and insights about pain and their life situation, through self-reflection in the solitary work in the Web-BCPA and through feedback from the health care professionals in MMR, was experienced as patient participation by the participants. Patient participation was described as structured ways to reach their goals of behavior change, which included analyzing resources and restrictions, problem solving, and evaluation. The individual’s emotional and cognitive resources and restrictions, as well as health care professionals and significant others’ attitudes and behavior influenced patient participation in the rehabilitation. To some extent there were experiences of restrained patient participation through the great content of the Web-BCPA.

**Conclusions:**

Patient participation was satisfactory in the Web-BCPA in combination with MMR. The combined treatment was experienced to increase patient participation in the rehabilitation. Being confirmed through self-identification and finding the content of the Web-BCPA trustworthy was emphasized. Patient participation was experienced as a learning process leading to new knowledge and insights. Higher user control regarding the timing of the Web-BCPA and therapist guidance of the content may further increase patient participation in the combined treatment.

## Introduction

Patient participation is a complex and multifactorial concept, and despite a large body of literature in the field, there is no consensus about a unifying definition that describes the concept [1-4]. To take part in or to be involved in one’s health care are fundamental definitions of patient participation [1,2,4,5]. The concept of patient participation may be applied to different areas of patient health care, such as the attending of treatments, decision making, and self-care [1,3,4]. In addition, there may be different implications of patient participation depending on the perspective in focus (patient, the health care organization, society) [1-3]. In the clinical health care meeting, patient participation can be described by the model of Patient-Centered Medicine (PCM) that includes understanding the patient as a whole person, acknowledging the patient’s expertise, shared decision making, and developing an ongoing therapeutic patient–health care professional relationship [6,7]. Health care professionals’ surrender of power and control to the patient has been considered an important aspect of patient participation [1]. Eldh et al [2] found that health care professionals had a narrower description of patient participation than the patients. Patient participation can influence treatment adherence and results, as well as health outcomes [8-10].

Patients with persistent pain have reported negative patient participation characterized by mistrust and dismissal from the health care professionals regarding their pain [11-14]. In contrast, patients with persistent pain in multimodal rehabilitation (MMR) have described positive patient participation built on mutual trust and respect in encounters with their health care professionals [15,16]. MMR is a recommended treatment for patients with persistent musculoskeletal pain [17-19]. It is based on a cognitive approach and a biopsychosocial explanation of persistent pain, and includes physical and psychosocial treatment components [17,19-21]. Activity in daily life and work is one main goal of MMR. The individual’s active participation in treatment sessions, as well as in setting goals, rehabilitation planning, and decision making is emphasized [16,21,22]. However, patients may have experiences of restrained patient participation in decision making due to the knowledge gap between the patient and the health care professionals, as well as the staff’s professional authority [1,6,7,15,23].

The entry of eHealth has had an impact on patient participation by increased access to health information, the extended delivery of health care, and a shift of power to the patient [24,25]. Web-based interventions have the potential to increase patient participation by enabling patients to play a more active role in their health care [25-28]. However, Web-based interventions suffer from nonusage attrition and low adherence [29-31]. It has been suggested that characteristics of the user, such as motivation, symptom panorama, and education level influence Web-adherence. In addition, Web-adherence is also related to the characteristics of the Web-based intervention, such as the flexibility of the program and how it is connected to specific personal needs of the user [30,31]. Altogether, there is a need of further research to find out how patients experience their participation in Web-based interventions [27,29].

In the county of Norrbotten, northern Sweden, the Web behavior change program for activity (Web-BCPA) was developed to propose an eHealth solution for a biopsychosocial treatment of persistent musculoskeletal pain. The Web-BCPA is a modified version of an existing Livanda Web-based program “To manage pain,” which had been developed in accordance with cognitive behavior therapy principles and focused on the individual’s active participation [32]. In cooperation with the founders of Livanda, “To manage pain” was revised with the aim to fit patients in an early stage of persistent pain. Altogether, the Web-BCPA aims to increase participants’ physical and cognitive activity in the rehabilitation and encourage activity in everyday life and work, including physical activity and promoting self-care [33]. There are a few studies that have reported on patient participation in Web-based interventions [30,34], and to our knowledge this is the first study of patient participation combining a therapist guided treatment with a self-guided Web-based intervention. Increased knowledge about what the Web-BCPA could add to patient participation in MMR may further illuminate the concept of patient participation in pain rehabilitation. Thus, the aim of this study was to explore patients’ experiences of patient participation in the Web-BCPA in combination with MMR in primary health care.

## Methods

### Study Design

A qualitative interview study was performed to obtain the variety of patients’ experiences of patient participation in the Web Behavior Change Program for Activity (Web-BCPA) in combination with MMR and to generate further knowledge of the topic [35,36]. The study was approved by the Regional Ethical Review Board of Umeå University, Sweden (Umu dnr 2011-383-31M).

### Informants and Selection

Informants eligible for the interview study were persons that participated in the randomized controlled trial (RCT) with the aim to investigate effects of the Web-BCPA for persistent pain in primary health care (NCT01475591), in the county of Norrbotten, Northern Sweden. They had been included to the RCT with the following inclusion criteria: (1) aged between 18 and 63 years; (2) persistent musculoskeletal pain with a duration of at least three months in the back, neck, shoulder, and/or generalized pain; (3) Örebro musculoskeletal pain screening questionnaire (ÖMPSQ) score ≥90, screening for psychosocial factors that indicates an estimated risk for long-lasting pain and future disability [37]; (4) work ability of at least 25 percent; (5) familiar with written and spoken Swedish; and (6) access to computer and Internet. Exclusion criteria were reduced cognitive ability or need of other health care. Additional inclusion criteria to this interview study were that the informants had spent at least 15 minutes per module in 5 of 8 modules in the Web-BCPA.

The study was performed from April 2012 to October 2014. Informants were consecutively included to the study in time for their 4-month follow-up of the RCT. Thirty-four persons were identified and informed about the interview study, and 22 persons gave their oral consent to be contacted through telephone calls by the interviewer for more details about the study. Three persons declined participation and finally 19 informants gave their written consent to participate. These included 15 women and 4 men, with mean age of 45 years. The majority (18 out of 19) lived with a spouse or partner, and about 50% (9/19) of the informants had children in the household. The informants’ educational level varied from elementary to university education. Furthermore, 63% (12/19) of the informants had permanent employment and 68% (13/19) were working at least 25% of a full time job or searching for work. The informants had musculoskeletal pain in the back, neck, shoulder, and/or generalized pain for in average 7.5 years. They rated mean pain intensity 67/100 on the visual analogue scale and had a mean ÖMPSQ score of 130 (Table 1).

**Table 1:** Informants’ characteristics.

Characteristics		Mean (range) or n (%)
Age in years, mean (range)		45 (27-60)
Woman, n (%)		15 (79)
Married or cohabit, n (%)		18 (95)
Children in the household, n (%)		9 (47)
**Education level^a^** **, n (%)**		
	Elementary	2 (11)
	Secondary	12 (63)
	University	5 (26)
**Working condition, n (%)**		
	Permanent or self-employed	12 (63)
	Temporary employment	3 (16)
	Unemployed	3 (16)
	Social benefits	1 (5)
Working^b^, n (%)		13 (68)
Pain duration in months, mean (range)		90 (5-156)
Pain intensity last 7 days^c^, mean (range)		67 (45-90)
ÖMPSQ^d^, mean (range)		130 (90-158)

^a^Elementary equals the first 9 years of education, secondary the following 3 years of education, and university represents all further education.

^b^Working at least 25% of a full time job or searching for work at time for baseline.

^c^The visual analogue scale. Score between 0 (no pain) to 100 (worst imaginable pain).

^d^The Örebro musculoskeletal pain screening questionnaire. Maximum score 210. A score ≥90 indicate a moderate estimated risk for long-lasting pain and future disability, and ≥105 indicates a higher estimated risk.

### Study Context

The informants received the Web-BCPA in combination with MMR.

The Web-BCPA was a self-guided Web-based intervention for pain management based on cognitive behavior therapy principles. The Web-BCPA consisted of 8 modules: (1) pain, (2) activity, (3) behavior, (4) stress and thoughts, (5) sleep and negative thoughts, (6) communication and self-esteem, (7) solutions, and (8) maintenance and progress. The modules contained information, assignments, and exercises, assimilated via educational texts, videos, and writing task. Assignments were interactive with the user. The Web-BCPA was delivered to the informant 1 module per week during the first 8 weeks of rehabilitation. The informants had access to the Web-BCPA 24/7 for 4 months. Administrative support in the Web-BCPA was provided, but there was no therapist guidance of the content. The Web-BCPA in detail has been described in a previous publication [33].

The informants spent 445 minutes (mean) in the Web-BCPA, with a range of 88 to 841 minutes. In total, 68% (13/19) of the informants had opened all 8 modules.

In the MMR, the patient and the team of health care professionals had drawn up an individualized rehabilitation plan. The rehabilitation plan included identification of the patient’s resources and restrictions, formulation of goals, planning of treatments, and dates for follow-up. The treatments were individual and/or in group sessions, and included for example physical activity, activity planning, symptomatic treatment, counseling, as well as home exercises. Further details about the MMR have previously been presented [33].

Each informant had between 7 and 36 (mean 18) treatment sessions. The informants had treatments by an occupational therapist (18 persons out of 19), physician (19 out of 19), physiotherapist (18 out of 19), and psychosocial counselor (15 out of 19). Nine informants had completed the MMR, and 10 continued their rehabilitation at time for the interview.

### Data Collection

Data was collected from qualitative interviews using a semistructured interview guide with open-ended questions. The interview began with an open question: “Please, tell me what patient participation is like for you?” The informants were then asked to describe their experiences of patient participation in the rehabilitation, the Web-BCPA combined with MMR. Each informant was interviewed once by the first author, within 1 month after the 4 months follow-up of the RCT. Eleven informants were interviewed at various health care centers in the county of Norrbotten, 7 persons at a conference-room at the county council building, and 1 person at home. The interviews were digitally recorded using an mp3 recorder, and ranged 31 to 56 (median value 48) minutes.

### Data Analysis

Data was analyzed using qualitative content analysis inspired by Graneheim and Lundman [38]. Content analysis is a systematic way to analyze the content in a text and qualitative content analysis includes latent interpretation of texts, which has been proven useful in many fields of research, for example health care sciences [38,39]. The researcher’s knowledge of the context of the study is important in the selection of informants, data collection, and data analysis [38].

First, the verbatim transcribed interviews were read several times to get an overall sense of the content. Then meaning units (words or sentences that are related to each other through their content and context) that answered to the aim of the study were marked. To shorten the text, the meaning units were condensed and labeled with a code. The codes were kept close to the text to keep the manifest expression of the text. The analysis was then copied into the freeware computer program Open Code [40].

Next step was to compare and compile the codes according to similarities and differences to create preliminary categories on a further abstraction level. The preliminary categories were compared against all data to construct definite categories, which were internally homogeneous and externally heterogeneous [38,41] A theme, which expressed the latent content, a thread of underlying meaning through the categories, developed during the analysis [38]. All authors participated in all steps of the analysis.

## Results

### Overview of Theme and Categories

The analysis of the informants’ experiences of patient participation in the Web-BCPA in combination with MMR resulted in 1 theme “It’s about me”, and 4 categories: “Take part in a flexible framework of own priority,” “Acquire knowledge and insights,” “Ways toward change,” and “Personal and environmental conditions influencing participation” (Textbox 1). The theme and categories are described in the following section, together with quotes from the informants.

**Results of the qualitative content analysis of informants’ experiences of patient participation in the Web Behavior Change Program for Activity (Web-BCPA) in combination with multimodal rehabilitation (MMR), presented with theme and categories.**

Theme:

- It’s about me

Category:

- Take part in a flexible framework of own priority
- Acquire knowledge and insights
- Ways toward change
- Personal and environmental conditions influencing participation

### It’s About Me

The theme “It’s about me” depicted patient participation as being confirmed in an available, flexible, and individualized framework of own choice. Informants’ experiences of being confirmed ran through all 4 categories and were expressed as patient participation in the interaction with the Web-BCPA and with the health care professionals in MMR. Being confirmed as a patient and as a person in one’s own team with many treatment options was experienced as a tailored rehabilitation. Though, the freedom of choice in the Web-BCPA entailed perceptions of restrained patient participation for some informants. A single situation of mistrust and disrespect with a health care professional in the MMR restrained patient participation but did not affect the overall perceptions of patient participation in the rehabilitation.

...it was obvious that it (the rehabilitation) was about me, it wasn’t about just anyone...it was about my problems, my strengths and how I felt...they (the health care professionals) started from a blank page, I was not fitted into an average template of how it ought to be...it (the rehabilitation) started with my point of view...

Interview 4, woman

To acquire knowledge and insights were thought of as patient participation, and included self-reflection, self-identification, and feedback. Informants experienced that being able to identify themselves with the content in the rehabilitation and finding it trustworthy were important to patient participation and being confirmed. Patient participation was described as their own process toward behavior change. Informants’ emotional and cognitive resources and restrictions, as well as health care professionals’ attitudes and behavior were important to patient participation.

### Take Part in a Flexible Framework of Own Priority

Within the category “Take part in a flexible framework of own priority,” patient participation was understood as taking part in a structured and flexible rehabilitation concept with opportunities to influence and a variety of treatments to choose according to one’s own needs and priorities.

...previously I had read about CBT (Cognitive Behavioral Therapy), but I had never thought of it as a help for my condition... I want to compare this rehabilitation with a smorgasbord from which is it easy to taste...

Interview 14, woman

Informants had experiences of patient participation in the solitary work in the Web-BCPA that included logging in, reading the texts, working with the assignments, and performing the exercises. Patient participation was emphasized by having access to the Web-BCPA on computer or tablet at all hours and locations. The opportunities to work in the Web-BCPA at home were experienced to provide continuity in the rehabilitation.

...thanks to the program (the Web-BCPA) I was able to perform the basic body awareness exercises of my own choice...and to repeat those that I felt most effective as many times that I preferred...the flexibility made it mine (the rehabilitation)...

Interview 4, woman

Although, some informants perceived restrained patient participation by the fact that they were not able to choose the starting time of the Web-BCPA course themselves (due to study protocol), as well as not being able to select a faster advancement in the program by themselves. Higher patient participation through participatory design of the content in Web-based interventions was suggested. Some informants experienced difficulties to choose area of interest in the Web-BCPA and that it became a burden to complete. In contrast, informants reported that they were supported by the health care professionals, including the rehabilitation coordinator, to make those choices in the MMR.

Patient participation was experienced as being part of one’s team with access to face-to-face meetings with health care professionals and available examinations and treatments through flexibility in treatment hours and timing. To simultaneously meet all health care professionals and significant others in dialogue and co-operation at team-conference meetings was emphasized. Also, the reasoning process between the health care professionals, and health care professionals reading and documenting in the patient records were perceived as patient participation.

...they (the health care professionals) were sensitive to understand me as a person...all of them...I felt very much involved when I met all health care professionals at the same time than when I met each at separate occasions...our decisions about the rehabilitation were mutual...

Interview 10, woman

Restrained patient participation was reported by the informants when a health care professional included in the patient’s rehabilitation but with clinical practice outside of the health care center did not attend the team-conference meeting. For example a physiotherapist of the private health care sector. Some informants experienced restrained patient participation when health care professionals decided to withdraw treatment with reference to that a patient’s symptoms were not severe enough.

Some informants thought of the rehabilitation concept similar to have a work or be in school, since participating entailed own efforts, to have something to contribute with and feeling satisfied. To some informants taking part in the Web-BCPA and the MMR was an integrated rehabilitation. Others described the Web-BCPA and the MMR as 2 parallel rehabilitations, which could entail different agenda.

### Acquire Knowledge and Insights

In the category “Acquire knowledge and insights,” patient participation was experienced as an interactive learning process toward knowledge and insights. Informants reported that gained knowledge and insights from working in the Web-BCPA strengthened their self-confidence and increased patient participation in the dialogue with health care professionals.

...my own thinking about my situation was confirmed by the content in the web-program (Web-BCPA)...this made me feel safe to share those thoughts (with the health care professionals) to acquire new knowledge that I can use in meetings with people that are involved in my rehabilitation...I was equipped with putting words on my thinking...

Interview 14, woman

...do you mean that it was easier to ask questions (to the health care professionals)?

Interview 14, interviewer

...yes...to be involved in my rehabilitation is much about me...to be confirmed by the content in the Web-program made me more powerful in meeting them (the health care professionals)...

Interview 14, woman

Patient participation was experienced by the informants as being able to identify themselves through the information and explanations about pain and symptoms, treatments, and advices given by the Web-BCPA content and the health care professionals. Self-identification was experienced to help informants to choose or exclude activities in the Web-BCPA. The informants found that there was a comparable message in the Web-BCPA and the MMR, and that “it was like made for them,” which increased trustworthiness and deepened knowledge and insights. Self-reflection and rehearsal was emphasized in the solitary work at a self-chosen work pace in the Web-BCPA, and experienced by the informants to favor learning and patient participation. Informants perceived that self-reflection was present to some extent in the contacts with health care professionals.

...working by myself in the Web-program made me reflect more and gave me insights, which I certainly passed on (to the team-members)...at the team-conference meetings there were more reasoning than reflection...

Interview 1, woman

Some informants described that new knowledge from the Web-BCPA developed into applied knowledge through feedback from a health care professional in the MMR. A continuous exchange of feed-back with health care professionals was emphasized in patient participation and in learning.

### Ways Toward Change

“Ways toward change” represented the informants’ experiences of patient participation in the Web-BCPA and MMR as ways to change one’s behavior. The informants’ experienced patient participation when they analyzed their situation taken into account their resources and restrictions, set goals for behavior change, and planned treatments and activities. Also, patient participation was stated when treatments, self-care, and planning were followed-up and evaluated. Awareness of improvements and goal attainment was perceived to favor patient participation and to motivate them to further actions for change. The informants stated that a written goal to strive for in the rehabilitation assured the change progress and patient participation. To adjust a goal or treatment planning in relation to progress or setback was described as patient participation.

...I feel it is important to set goals and to follow-up those goals...and to understand why a goal is reached and why another is not...this made me aware of that I needed other tools (in the rehabilitation)...

Interview 14, woman

Informants described that they guided themselves in their ways toward change in the Web-BCPA and that problem solving was emphasized. Some informants experienced restrained patient participation through difficulties to come up with a problem area. Patient participation was reported when informants monitored results shown by the interactive graphs in the Web-BCPA.

...days when I had a lot of pain I used to remain sedentary, and as soon as I had a better day I was eager to do all kinds of activities that day...before I started the assignment activity planning (in the Web-BCPA) I was not aware of how my behavior related to the days with pain, but by monitoring this over time I started to plan my daily activities in a more balanced way...

Interview 11, woman

In the MMR, informants experienced that drawing up a rehabilitation plan in mutual agreement with health care professionals was ways to behavior change. Some informants emphasized patient participation as having their own choice to play an active role in rehabilitation planning by contributing a lot in decision-making with own preferences and own suggestions. Others experienced patient participation as having a choice to play a more passive role by responding to and considering the health care professionals’ opinions. A development to play a more active role in rehabilitation planning with time was reported. There were reports that patient participation and the change process benefited from choosing the same problem area in the Web-BCPA and in the MMR. Some informants experienced that the change process proceeded through new behavior and motivation even though they had completed their rehabilitation.

### Personal and Environmental Conditions Influencing Participation

Informants talked about various conditions related to the rehabilitation framework that influenced patient participation in the rehabilitation. They described emotions and cognitions that affected patient participation. Having motivation, interest, commitment, and self-confidence were perceived to favor patient participation. In addition, some informants stated that their work experience, such as having a solution-focused work, or to enjoy working at the computer, facilitated patient participation in the rehabilitation.

...I feel that one has to be motivated to participate in the course (the Web-BCPA) since it requires that I set aside time to log in to the program several times a day...it takes time to read all the texts and to do the assignments...

Interview 13, woman

Pain, fatigue, and other psychological symptoms were perceived to limit patient participation. Some informants experienced that having such symptoms restrained participation more in the Web-BCPA than in the MMR. On the other hand, informants described that the Web-BCPA provided opportunities to rehabilitation during periods with severe symptoms without having to be present at the health care center. In addition, perceiving lack of knowledge in medical issues and treatments was experienced to restrain patient participation.

Previous experiences of a positive therapeutic relationship with a health care professional in the team were perceived to facilitate patient participation in the MMR. Awareness of a health care professional’s stressful work situation and limited health care resources were stated by the informants to restrain patient participation. Support, trust and respect from a family member, employer, the Swedish Social Insurance Agency (SSIA) or the Employment Service were experienced to facilitate patient participation in the rehabilitation. Some informants experienced that demands on return to work of the SSIA entailed stress and fatigue and restrained patient participation and caused setbacks in the rehabilitation.

...I planned to complete the program (the Web-BCPA)...I am not sure how much I had left...probably the last module...but I was denied sick-leave compensation by the Social Insurance Agency and had to put in a lot of energy to explain my situation and meet with the psychosocial counselor...I did not have the strength to do anything else...I have used so much energy to fight for my cause...

Interview 8, man

## Discussion

### Principal Findings

Patient participation in the Web-BCPA in combination with MMR was explored in this study. All informants had experiences of satisfying patient participation. The comprehensive theme “It’s about me” revealed patient participation as an individualized and empowered interaction with the Web-BCPA and with health care professionals within the MMR, a rehabilitation the informants perceived as their own. Our findings showed that informants’ perceptions of being confirmed were fundamental to patient participation. The importance of being confirmed in the patient–health care interaction has previous been reported [14,15,42,43]. However, findings that informants experienced being confirmed through the solitary work in the Web-BCPA implicate new knowledge to patient participation. They described that they were confirmed when they could identify their illness experience and life situation, as well as their own thoughts and cognitions about their pain condition, in the texts and the assignments of the Web-BCPA. There were many implications to being confirmed and the informants perceived this fundamental to other experiences of patient participation in the rehabilitation, such as the gain of knowledge and insights, and behavior changes. In addition, perceptions of being confirmed entailed the informants’ experiences of a trustworthy and comparable message in the Web-BCPA and in the MMR.

The informants described that gained knowledge and insights from the Web-BCPA increased their self-confidence and empowered them in the dialogue with health care professionals. Previous research has showed that patients wish to play a more active role in decision making in their MMR but the lack of knowledge in medical issues and treatment options restrained them [15]. To narrow the knowledge gap between the patient and the health care professional has been reported as an important factor to increase patient participation and to improve the cooperation [1,2]. Our findings indicate that the Web-BCPA can be a useful tool in narrowing the knowledge gap.

Acquiring knowledge and insights was both experienced as patient participation and described as means to increase patient participation. The informants perceived that the solitary work in the Web-BCPA had an important role to acquire knowledge and insights by providing opportunities for self-reflection and rehearsal. Such internal cognitive processes are known to reinforce and modify learning [44,45]. In contrast, the informants reported less self-reflection in the MMR. On the other hand, informants emphasized the feedback in meetings with health care professionals in MMR, which was experienced to facilitate applied knowledge. Feedback from health care professionals has been shown to support an individual’s behavior change [45-47]. Some informants made a clear statement that patient participation in the MMR was the effective treatment in the rehabilitation for behavior change. Other informants gave examples of successful problem solving in the Web-BCPA that led to behavior change. This is in line with participants’ experiences of behavior change as increased engagement in physical and social activity after taking part in a Web-based intervention with mindfulness-based cognitive therapy aimed to reduce depressive symptoms [48].

An overall interpretation of our data that we find interesting to discuss and which may inspire to further research regarding patient participation, was a distinction between “taking part” and “participating” in the rehabilitation. Some informants described patient participation as having attended meetings with health care professionals and having had treatments, more on an operational level of adherence, without further reflections on the emotional or cognitive processes that may be involved in patient participation. In contrast, other informants’ talked a lot about their emotional and cognitive experiences in relation to patient participation, such as feelings, reflections, and appraisal. These various perceptions of patient participation could be important not only to patients’ experiences of patient participation, but also to treatment adherence and outcomes. Some informants that reported on having reached a goal or having been successful in behavior change, talked about this in relation to emotional and cognitive processes, such as awareness and insights. In line with Herlitz et al [47], emotional feedback from health care professionals to enhance a patient’s emotional and cognitive relation to their rehabilitation, may be important to ensure adherence and positive outcomes. It may not be sufficient to only attend the treatments.

The informants’ experiences of patient participation in the Web-BCPA in combination with the MMR had much in common with PCM [7,49,50], which is considered to be a key element of high-quality health care [51,52]. Informants perceived the combined treatment as a personalized and customized rehabilitation. Lyden et al [53] found similar consistency with the PCM model among participants in a Web-based intervention designed to promote weight loss through healthy eating and physical activity. The participants reported the Web-based intervention as individualized with opportunities to make their own decisions [53]. Furthermore, our findings may add to the PCM model that an individual’s learning process and the acquiring of knowledge and insights might need to be included in the model. It may not be sufficient to acknowledge a patient’s present expertise of their illness experience and their life situation. By increasing knowledge and insights about pain and cognitive skill processes, patient participation in the rehabilitation can improve.

### Strengths and Limitations

We included women and men of various ages and from different health care centers in the county of Norrbotten, to collect a variety of experiences which may have increased credibility. The informants’ experiences of patient participation in the Web-BCPA in combination with the MMR were based on the interaction with the Web-BCPA, as well as team-conference meetings and individual meetings with nurses, occupational therapists, physicians, physiotherapists, psychologists, psychosocial counselors, and rehabilitation coordinators. One limitation with the selection of participants was that patients that had spent less time in the Web-BCPA were excluded from this study, which may have positively influenced the results. However, the objective of this study was to explore patients’ experiences of patient participation in the Web-BCPA, and therefore we decided that to have such experiences, the participant needed to have had the chance to assimilate some of the content of the Web-BCPA. We set a lower limit of time spent in the Web-BCPA to 75 minutes, and that the informants should have opened 5 modules out of 8.

To increase the credibility of the findings, all authors participated in the data analysis, which was performed with care. All researchers had a professional background in physiotherapy and 1 was also a psychologist, which may have influenced the analysis. Meaning units and codes were kept close to the text, which may have reduced the risk of misleading interpretations. During analysis, all authors reflected on and discussed codes, categories, and theme until consensus was obtained. Our results are 1 possible description of patient participation in the Web-BCPA in combination with the MMR. The 4 categories are not totally exclusive as the theme ran through all categories, and it is not always obvious when an experience belongs to 1 category or another since human experiences are intertwined [38].

Data was collected by performing interviews consecutively over the whole RCT time period of 2 years. This may imply both an advantage and a disadvantage. The advantage is that experiences of patient participation were captured as the project developed at the health care centers. The disadvantage is the risk of inconsistency when data collection extends over time, and the interviewer may acquire new insights with time [38]. To increase dependability, an interview guide was used which gave all informants the same opportunities to contribute with their experiences. The interviews were rich and contained detailed descriptions of experiences of positive and negative patient participation.

We consider that part of our results may be transferable to patients with persistent pain in comparable multimodal rehabilitation in primary health care, as well as to other team rehabilitation using a cognitive approach. There is a limited transferability of our results to patients’ interaction with similar self-guided Web-based interventions since the treatment was given in combination with MMR.

### Conclusions

Patient participation in the Web-BCPA in combination with MMR was experienced as personal confirmation “It is about me,” where it was possible to take part in a rehabilitation framework of one’s own priority and have the opportunity to influence. Being confirmed was emphasized in the interaction with the Web-BCPA and with health care professionals in the MMR. Patient participation was to acquire knowledge and insights and to find ways to behavior change. In the Web-BCPA, the solitary work and self-reflection were stated as patient participation. Dialogue and feed-back from health care professionals were emphasized in the MMR. The combined treatment was experienced to increase patient participation in the rehabilitation. Although, not being able to fully control the administration of the Web-BCPA, as well as having difficulties to choose from its content, were experienced to restrain patient participation.

## Abbreviations

MMR: multimodal rehabilitation
ÖMPSQ: Örebro musculoskeletal pain screening questionnaire
PCM: Patient-Centered Medicine
RCT: randomized controlled trial
Web-BCPA: Web Behavior Change Program for Activity
